# Optimizing first-line therapy for advanced *EGFR*-mutated NSCLC: a patient-centered approach for low- and middle- income countries

**DOI:** 10.3389/fonc.2026.1940079

**Published:** 2026-10-21

**Authors:** Ana Caroline Zimmer Gelatti, Eldsamira Mascarenhas, Guilherme Harada, Sofia Vidaurre Mendes, Vladmir Claudio Cordeiro de Lima

**Affiliations:** 1 Grupo Brasileiro de Oncologia Torácica, Porto Alegre, Brazil; 2 Grupo Oncoclínicas, Porto Alegre, Brazil; 3 Hospital São Lucas Pontifícia Universidade Católica do Rio Grande do Sul (PUC/RS), Porto Alegre, Brazil; 4 D’Or Oncology, São Paulo, Brazil; 5 D’Or Institute for Research and Education (IDOR), Rio de Janeiro, Brazil; 6 Oncology Center, Hospital Sírio-Libanês, São Paulo, Brazil; 7 Thoracic Cancer Reference Center - A. C. Camargo Cancer Center, São Paulo, Brazil

**Keywords:** decision making, EGFR, first-line therapy, metastasis, non-small cell lung cancer

## Abstract

The management of advanced *EGFR*-mutated non-small cell lung cancer (NSCLC) has evolved from limited targeted options to multiple effective first-line strategies, improving survival but increasing toxicity and treatment burden. This narrative review synthesizes evidence on first-line therapies, integrating clinical outcomes, toxicity profiles, and patient-centered considerations. Recent advances introduce trade-offs between efficacy and tolerability. Patients often prioritize symptom control, functional status, and time outside the healthcare system over modest gains in survival. Treatment selection must account for adverse events, monitoring requirements, and multidisciplinary care. In settings with limited access to molecular testing and novel therapies, such as Brazil and other middle-income countries, disparities further influence decisions. Optimal treatment requires balancing efficacy with safety, feasibility, and patient preferences; therefore, shared decision-making is essential for aligning clinical benefit with real-world constraints and enabling personalized care.

## 1 Introduction

Non-small cell lung cancer (NSCLC) accounts for 80-85% of cases of lung cancer, affecting ~1.98 - 2.11 million people worldwide (1). Epidermal growth factor receptor gene (*EGFR*) mutations are present in 32.3% of the world’s NSCLC patients (2). *EGFR-*mutated NSCLC represents a distinct clinical and biological subset, more frequently observed in women, never-smokers, and patients of Asian ancestry, and is characterized by a high propensity for central nervous system (CNS) metastases, reflecting a more aggressive disease course with unique patterns of dissemination (3, 4). Additionally, a large proportion of NSCLC patients are diagnosed at advanced stages, including metastatic (stage IV) disease, and current estimates suggest that around 40% of NSCLC patients present with distant metastases at diagnosis. The 5-year survival rate for metastatic *EGFR*-mutated NSCLC patients is estimated to be very low (11 to 19%) (5, 6).

The availability of molecular testing to identify specific *EGFR* mutations and the development of novel therapies targeting them have shifted the paradigm for the management of advanced *EGFR*-mutated NSCLC. These new therapies, while offering promising improvement in progression-free survival (PFS) and overall survival (OS), also introduce a broader spectrum of toxicities, necessitating a more complex approach to patient care. They lead to careful consideration of various treatment options, their respective efficacy and safety profiles, different lines of management, including prophylaxis and adverse event handling, and the strategic sequencing of subsequent therapies. Concurrently, there is growing recognition in the literature of the importance of incorporating patient perspectives and preferences into therapeutic choices.

In Brazil, the management of advanced *EGFR*-mutated NSCLC is characterized by profound disparities between the private healthcare system and the public health system (SUS). While the Brazilian Health Regulatory Agency (ANVISA) has approved next-generation treatments, the public sector (SUS) faces significant barriers. The National Commission for Technology Incorporation in SUS (CONITEC) has not yet approved third-generation TKIs or intensive combinations for first-line therapy; consequently, public health system patients remain largely restricted to first-generation EGFR-TKIs as their only accessible targeted option. Conversely, access is substantially greater in the private sector but remains heterogeneous, as well as combinations, as coverage is regulated and mandated by the National Supplementary Health Agency (ANS).These diagnostic and therapeutic barriers directly impact clinical outcomes and highlight the necessity of implementing feasible and sustainable care strategies adapted to regional healthcare realities (7).

Within this evolving landscape and intrinsic challenges, this narrative review aims to synthesize critical information and best practices regarding current first-line treatments for advanced NSCLC with common *EGFR* mutations (exon 19 deletion or L858R), encompassing efficacy, safety, and patient-centered considerations. It also aims to support therapeutic decision-making and patient-centered care, with particular attention to challenges faced in Brazil and other low- and middle-income countries. Biologically distinct molecular subgroups, such as *EGFR* exon 20 insertions and atypical mutations, follow fundamentally different treatment algorithms and are outside the scope of this review.

To identify relevant publications, we conducted a comprehensive search of PubMed/MEDLINE and SciELO (Scientific Electronic Library Online) databases for articles published up to June 2026. The search terms and key concepts included: “*EGFR* mutation”, “non-small cell lung cancer”, “NSCLC”, “first-line therapy”, “osimertinib”, “amivantamab”, “lazertinib”, “FLAURA”, “MARIPOSA”, “patient preferences”, “treatment barriers”, and “Brazil”. Only peer-reviewed, English-language publications, including phase III clinical trials, secondary analyses, consensus guidelines, regulatory documents (FDA, ANVISA), and patient-preference studies, were considered for selection. The final selection was based on relevance to the clinical decision-making process for first-line systemic treatment of advanced *EGFR*-mutated NSCLC.

## 2 *EGFR*-mutated NSCLC therapeutics, regulatory status (FDA and ANVISA), and current guidelines

Gefitinib (AstraZeneca) (8) and erlotinib (Genentech/Roche) (9) were the first-generation EGFR-targeting TKIs approved by the US Food and Drug Administration (FDA) in 2003 and 2004 and by the Brazilian Agência Nacional de Vigilância Sanitária (ANVISA) in 2010 and 2006, respectively. Despite their efficacy against the most common *EGFR* mutations, exon 19 deletions and L858R, present in over 80% of *EGFR*-mutated NSCLC patients, these drugs displayed low effectiveness against rare resistance mutations, such as T790M, and mutations that reduce conventional TKI sensitivity, such as exon 20 insertion. Additionally, first-generation drugs demonstrated reduced efficacy in patients with CNS metastases, which partially contributed to the development of second-generation drugs, afatinib (Boehringer Ingelheim) (10) and dacomitinib (Pfizer) (11). These were approved by the FDA in 2013 and 2018, respectively. ANVISA approved afatinib in 2016; however, dacomitinib is not registered by ANVISA (**Figure 1**).

**Figure 1:**
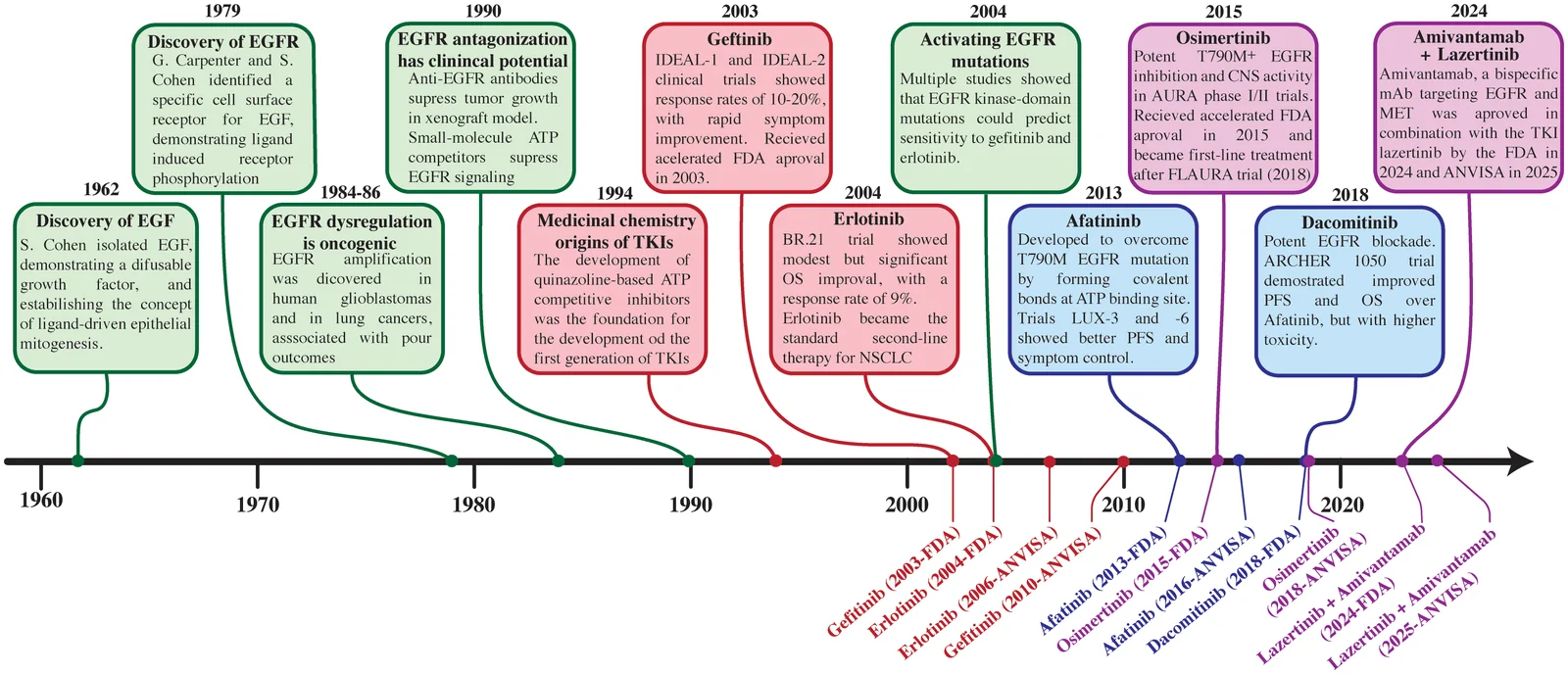
Timeline of TKI development and approval by the FDA and ANVISA.

The currently FDA-approved third-generation TKIs, osimertinib (AstraZeneca) (12) and lazertinib (Johnson & Johnson) (13), were developed to achieve increased CNS penetration and efficacy against the T790M mutation, thereby targeting metastatic tumors. Lazertinib was approved by the FDA in combination with amivantamab in 2024 and registered by ANVISA in 2025. Osimertinib was approved by the FDA in 2015 and ANVISA in 2018. It is a mutant-selective EGFR inhibitor with good results against exon 19 deletions and L858R mutations, and has demonstrated high CNS penetration. Since 2018, osimertinib has been the standard first-line therapy for *EGFR*-mutated NSCLC.

In addition to TKIs, monoclonal antibodies play an important role in the treatment of *EGFR*-mutated NSCLC. In 2021, amivantamab (Janssen/Genmab) was granted accelerated approval by the FDA for patients with metastatic NSCLC with *EGFR* exon 20 insertion mutations (14), which converted to full approval in 2024, when amivantamab in combination with chemotherapy (carboplatin and pemetrexed) became the first-line treatment for locally advanced or metastatic NSCLC with *EGFR* exon 20 insertion mutations.

According to the most recent National Comprehensive Cancer Network (NCCN) guidelines (15), upon diagnosis of any histologic subtype of locally advanced or metastatic NSCLC, patients must undergo multigene panel biomarker testing to identify potentially actionable mutations. Once *EGFR* exon 19 deletion or L858R mutation is detected, regardless of being discovered before or during first-line systemic therapy, NCCN preferentially recommends the use of the following drugs as first-line specific therapy: i) osimertinib monotherapy; ii) osimertinib combined with pemetrexed and carboplatin or cisplatin; and iii) lazertinib in combination with amivantamab.

This review focuses on summarizing clinical trial data for NCCN-recommended treatments, emphasizing efficacy and safety while incorporating patient-centered considerations, such as time in care, the need for additional prophylaxis, and involvement of other medical specialties, to support informed physician–patient shared decision-making.

## 3 Efficacy

### 3.1 Osimertinib monotherapy (FLAURA)

Osimertinib monotherapy was evaluated in patients with locally advanced or metastatic *EGFR*-mutant disease harboring exon 19 deletion or L858R and was compared with first-generation EGFR-TKIs in the FLAURA trial (12). FLAURA was designed as a double-blind study that randomized 556 treatment-naïve patients diagnosed with metastatic *EGFR*-mutated non-squamous NSCLC into 2 groups (1:1 ratio) to receive either osimertinib (80 mg once daily) or a first-generation EGFR-TKI (gefitinib at 250 mg once daily or erlotinib at 150 mg once daily) orally.

Osimertinib significantly improved PFS and OS when compared with first-generation EGFR-TKIs, with a median PFS of 18.9 months *vs.* 10.2 months with first-generation EGFR-TKIs, with an HR (95% [CI]) of 0.46 (0.37–0.57) and median OS of 38.6 months *vs.* 31.8 months with first-generation EGFR-TKIs with an HR of 0.80 (0.64–1.00) (**Figure 2**).

**Figure 2:**
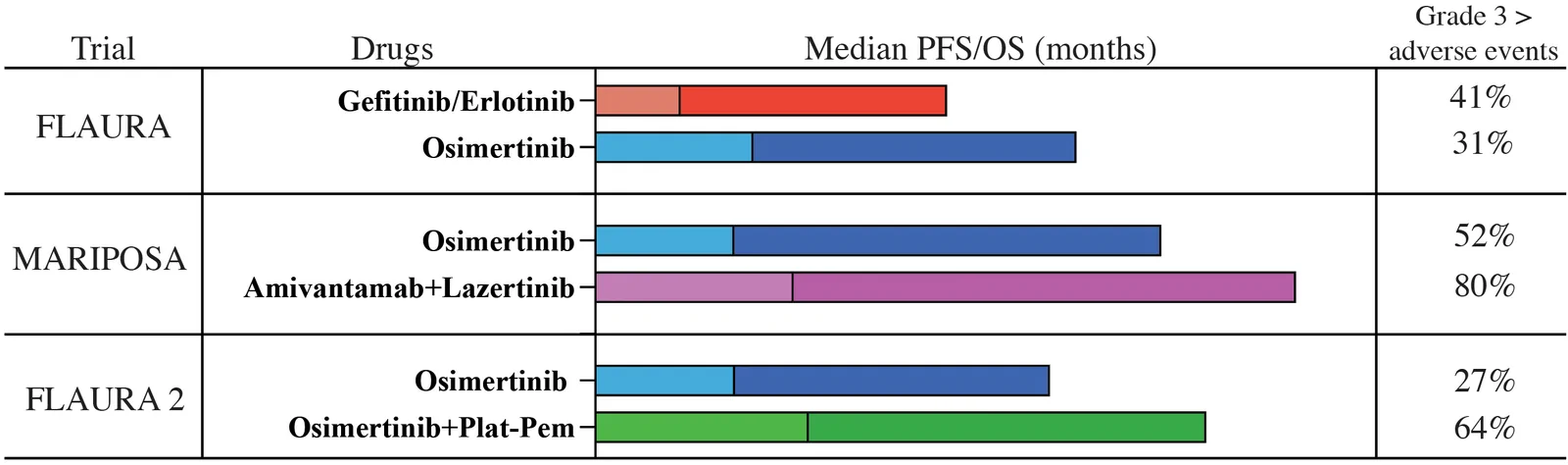
Summary of efficacy outcomes from first-line *EGFR*-mutated NSCLC trials and the rate of grade 3 or above adverse events. Median PFS and OS, and the rate of grade 3 or above adverse events in the FLAURA, MARIPOSA, and FLAURA 2 trials are shown. *MARIPOSA Grade 3 or above adverse event rates are updated to reflect mature safety analyses (amivantamab plus lazertinib: ~80% vs. osimertinib: ~52%, compared to initial reports of 75% vs. 43%). Overall survival data for MARIPOSA also reflect mature 3-year observed results (3-year OS rate 60% vs. 51%, HR 0.75)* (18).

Intracranial efficacy was a key endpoint, as CNS metastases are of special concern in EGFR-mutated NSCLC and 19% of the study population presented with CNS metastases at baseline (11, 16). In the subgroup of patients with baseline CNS metastases, osimertinib markedly improved PFS compared with earlier-generation TKIs, with a median CNS PFS of 15.2 months *vs.* 9.6 months (HR 0.48; 95% CI, 0.34–0.69) (16). Among patients with measurable baseline CNS lesions, the CNS objective response rate (ORR) was substantially higher with osimertinib than with first-generation EGFR-TKIs (91% *vs.* 68%, respectively) (16). Furthermore, the median OS in patients with CNS metastases was 38.6 months with osimertinib compared with 29.6 months with first-generation TKIs (HR 0.82; 95% CI, 0.53–1.26) (16).

### 3.2 Amivantamab plus lazertinib (MARIPOSA)

In the MARIPOSA trial, amivantamab combined with lazertinib was compared with osimertinib in patients with locally advanced or metastatic NSCLC harboring common activating EGFR mutations, specifically exon 19 deletions and L858R substitutions (17). In this trial, 1074 previously untreated patients were randomly assigned in a 2:2:1 ratio to receive lazertinib + amivantamab, osimertinib only or lazertinib only. Amivantamab was administered IV weekly at a dose of 1050 mg (or 1400 mg in patients with a body weight of ≥80 kg) for the first 4 weeks (cycle 1), with the first infusion split over a period of 2 days (with 350 mg given on cycle 1 day 1, and the remainder given on cycle 1 day 2). Starting at cycle 2, the same amivantamab dose was administered every 2 weeks. Osimertinib (80 mg) and lazertinib (240 mg) were administered orally daily.

The combination of amivantamab and lazertinib significantly improved PFS and OS when compared with osimertinib monotherapy. Median PFS was 23.7 months vs. 16.6 months with amivantamab + lazertinibe compared to osimertinib, with a hazard ratio of 0.70 (0.58–0.85). Updated overall survival analysis showed a statistically significant benefit for amivantamab plus lazertinib compared to osimertinib, with a 3-year OS rate of 60% vs. 51%, respectively (HR 0.75; 95% CI, 0.61–0.92; p=0.005) (18) (**Figure 2**).

It is important to highlight that amivantamab plus lazertinib also displayed improved PFS over osimertinib in high-risk subgroups of patients with detectable circulating tumor DNA (20.3 *vs.* 14.8 months; HR 0.68 [0.53-0.86]; p=0.002), *TP53* co-mutations (18.2 *vs.* 12.9 months; HR 0.65 [0.48-0.87]; p=0.003) and liver metastasis (18.2 *vs.* 11.0 months; HR 0.58 [95% CI 0.37-0.91]; p=0.017) (15, 17). For comparison, a recent study analyzing data from 1323 patients (95% presenting at least one high-risk factor) treated with osimertinib as monotherapy, drawn from medical oncology databases in the United States, found a real-world (RW) median OS of 28.6 months (95% confidence interval, 26.8–30.9) (18).

CNS control was also a key therapeutic target and 30% to 40% of the MARIPOSA population had baseline CNS metastases (16, 17). The dual EGFR/MET blockade reduced the risk of CNS progression or death by approximately 42% compared with osimertinib (HR 0.58; 95% CI, 0.44–0.78) (19). The median CNS PFS was not reached in the combination group, whereas it was 16 to 18 months in the osimertinib monotherapy group, while the CNS ORRs were high and comparable between both treatment arms (approximately 83–85% with the combination *vs.* 80–82% with osimertinib) (19).

### 3.3 Osimertinib plus platinum-pemetrexed chemotherapy (FLAURA 2)

The FLAURA 2 trial compared osimertinib plus chemotherapy with osimertinib monotherapy (20). This open-label trial randomized 557 patients with *EGFR*-mutated (exon 19 deletion or L858R mutation) advanced NSCLC who had not previously received treatment into 2 groups (1:1 ratio). One group received osimertinib (80 mg once daily) plus intravenous pemetrexed (500 mg per square meter of body-surface area) and cisplatin (75 mg per square meter) or carboplatin (AUC 5) every 21 days for four cycles, followed by maintenance with pemetrexed and osimertinib. The second group received oral osimertinib (80 mg daily) continuously.

Investigators found that osimertinib plus chemotherapy significantly improved PFS and OS compared with osimertinib monotherapy. Median PFS was 25.5 months *vs.* 16.7 months with osimertinib monotherapy (HR 0.62; 95% CI, 0.49–0.79, p<0.001), and median OS was 47.5 months *vs.* 37.6 months (HR 0.77; 95% CI, 0.63–0.95, p=0.02) (**Figure 2**). In the subgroup of patients with CNS metastases, median PFS was 24.9 months with osimertinib plus chemotherapy *vs.* 13.8 months with monotherapy (HR 0.58; 95% CI, 0.44–0.78) (20, 21). Preliminary data from the TOP study, which evaluated osimertinib with or without chemotherapy in patients harboring concurrent *TP53* mutations, found a median PFS of 34 months for combination therapy and 15.6 months for monotherapy (HR 0.44; 95% CI, 0.32-0.6; p<0.001) and a trend for OS benefit (HR 0.57, 95% CI, 0.38-0.88) with safety profiles similar to those of FLAURA 2, supporting the robustness of the treatment effect (21).

The addition of chemotherapy reduced the risk of CNS progression or death, with a reported HR of approximately 0.58 compared with osimertinib monotherapy. Median CNS PFS was not reached in the combination arm and was approximately 24 months in the osimertinib-alone arm at the time of reporting. CNS ORRs were high in both groups (osimertinib + platinum/pemetrexed: ~90%; osimertinib monotherapy: ~85%), but not statistically different. Following the publication of the FLAURA2 trial, the combination of osimertinib with platinum–pemetrexed chemotherapy was approved by the Brazilian Health Regulatory Agency (ANVISA) in April 2024 for the first-line treatment of patients with locally advanced or metastatic NSCLC harboring *EGFR* exon 19 deletions or exon 21 L858R mutations (20).

Collectively, these data demonstrate that osimertinib markedly improves intracranial outcomes over earlier-generation TKIs and that both chemotherapy intensification (FLAURA2) and dual EGFR/MET blockade (MARIPOSA) further delay CNS progression relative to osimertinib monotherapy. However, toxicity must be weighed against efficacy in individualized treatment decisions.

## 4 Safety

### 4.1 Osimertinib monotherapy (FLAURA)

In the FLAURA trial, adverse events occurred in 89% of patients in both groups. Grade 3 or above adverse events were reported in 31% of patients treated with osimertinib and 41% of those treated with first-generation EGFR-TKIs (**Figure 2**). The most reported adverse events were rash or acneiform dermatitis (58% in the osimertinib group and 78% in the first-generation EGFR-TKI group), diarrhea (58% and 57%, respectively), and dry skin (36% in each group). Cardiac effects (changes in QT interval) were more frequent in the osimertinib group (10%) than in the first-generation EGFR-TKI group (5%). In the FLAURA trial, adverse events leading to dose reduction occurred in 13% of patients in the osimertinib group and 18% in the first-generation EGFR-TKI group, while therapy discontinuation occurred in 4% and 5% of patients, respectively, and treatment-related deaths were observed in less than 1% of patients in both groups (12) (**Table 1**).

**Table 1:** Rate of adverse events in patients from first-line *EGFR*-mutated NSCLC trials.

	FLAURA				MARIPOSA				FLAURA 2			
	Gefitinib/Erlotinib		Osimertinib		Osimertinib		Amivantamab+Lazertinib		Osimertinib		Osimertinib+Chemotherapy	
Event description	All	Grade 3>	All	Grade 3>	All	Grade 3>	All	Grade 3>	All	Grade 3>	All	Grade 3>
Alanine aminotransferase elevation	27	9	6	0.5	13	2	36	5	8	0	20	1
Alopecia	13	0	7	0	0	0	0	0	0	0	0	0
Anemia	9	1	12	1	21	2	23	4	22	0.5	46	20
Aspartate aminotransferase elevation	25	4	9	1	14	1	29	3	5	0.5	17	0.5
Asthenia	0	0	0	0	11	1	19	3	0	0	0	0
Blood creatine increase	0	0	0	0	0	0	0	0	4	0	17	0
Constipation	13	0	15	0	13	0	29	0	10	0	29	5
Cough	15	0.5	16	0	21	0	15	0	0	0	0	0
Decreased appetite	19	2	20	3	18	1	24	1	9	1	31	3
Diarrhea	57	2	58	2	44	1	29	2	41	0.5	43	3
Dry skin	36	1	36	0.5	14	0	16	0	24	0	18	0
Dyspnea	7	1	13	0.5	16	4	12	1	0	0	0	0
Fatigue	12	0	14	1	10	1	17	1	9	0.5	28	3
Headache	7	0	12	0.5	0	0	0	0	0	0	0	0
Hypoalbuminemia	0	0	0	0	6	0	48	5	0	0	0	0
Hypocalcemia	0	0	0	0	8	0	21	2	0	0	0	0
Infusion-related reaction	0	0	0	0	0	0	63	6	0	0	0	0
Leukopenia	0	0	0	0	15	0	6	0.5	7	0.5	16	3.5
Muscle Spasms	0	0	0	0	7	0	17	0.05	0	0	0	0
Nausea	19	1	14	0	14	0.5	21	1	10	0	43	1
Neutropenia	0	0	0	0	0	0	0	0	3	1	25	11
Pain in extremity	0	0	0	0	5	0	15	0.5	0	0	0	0
Paronychia	33	1	35	0.5	28	0.5	68	11	27	0.5	24	1
Peripheral edema	0	0	0	0	6	0	36	2	4	0	15	0
Prolonged QT interval on ECG	4	1	10	2	0	0	0	0	0	0	0	0
Pruritus	16	0	17	0.5	17	0	24	0	0	0	0	0
Pulmonary embolism	0	0	0	0	5	2	17	8	0	0	0	0
Pyrexia	4	0.5	10	0	0	0	0	0	0	0	0	0
Rash or acne	78	7	58	1	31	1	62	31	21	0	28	0.5
Stomatitis	20	0.5	29	1	21	0	29	0	18	0	25	0.5
Thrombocytopenia	0	0	0	0	20	1	16	0.5	4	1	18	7
Upper respiratory	6	0	10	0	0	0	0	0	0	0	0	0
Vomiting	10	1	11	0	0	0	0	0	6	0	26	1

The numbers represent the percentage of patients who had any adverse event, or grade 3 or above adverse events, during the clinical trials.

### 4.2 Amivantamab plus lazertinib (MARIPOSA)

In the MARIPOSA trial, most patients experienced at least one adverse effect (100% with amivantamab plus lazertinib *vs.* 99% with osimertinib). Grade 3 or above adverse events were reported in approximately 80% of patients treated with amivantamab plus lazertinib and 52% of those treated with osimertinib (**Figure 2**) (17, 20). Infusion-related reactions occurred in 63% of the patients treated with amivantamab + lazertinib. Venous thromboembolic adverse events were reported in 37% of patients in the amivantamab + lazertinib group and in 9% of those in the osimertinib group. Other adverse events of grade ≥3 were more frequently reported in the amivantamab plus lazertinib group: paronychia (11% *vs*. <1%), rash (15% *vs.* 1%), and acneiform dermatitis (8% *vs.* 0%). For amivantamab + lazertinib, events leading to interruption, dose reduction, or complete discontinuation of any agent were reported in 83%, 59%, and 35% of patients, respectively, while for osimertinib, these events occurred in 39%, 5%, and 14% of patients, respectively. Treatment-related deaths were observed in 3% of amivantamab + lazertinib-treated patients and less than 1% of osimertinib-treated patients (19) (**Table 1**).

### 4.3 Osimertinib plus platinum-pemetrexed chemotherapy (FLAURA 2)

In the FLAURA 2 trial, most patients experienced at least one adverse effect (100% with osimertinib plus chemotherapy *vs*. 97% with osimertinib monotherapy). Adverse events of grade 3 or above were reported in 64% of patients in the osimertinib + chemotherapy group and in 27% of patients in the osimertinib group (**Figure 2**). Grade 3 or above adverse events that were more frequently reported with osimertinib plus chemotherapy *vs.* osimertinib monotherapy are worth mentioning: neutropenia (14% *vs.* 1%) and thrombocytopenia (7% *vs.* 1%), which are early hematologic toxicities predominantly observed during the induction phase of treatment. Adverse events leading to the discontinuation of one or more drugs were observed in 6% of osimertinib-treated patients and 48% of osimertinib + chemotherapy-treated patients (mostly chemotherapy discontinuation), while treatment-related deaths occurred in less than 1% of osimertinib-treated patients and in approximately 2% of osimertinib + chemotherapy-treated patients (22) (**Table 1**).

Taken together, these safety profiles underscore that while third-generation EGFR-TKI monotherapy has a highly manageable toxicity profile (12), both chemotherapy intensification (FLAURA 2) (22) and dual EGFR/MET blockade (MARIPOSA) (19) significantly increase the incidence of severe (Grade ≥ 3) adverse events, cumulative treatment complexity, and therapy discontinuation rates. Therefore, preemptive prophylaxis, diligent multidisciplinary monitoring, and patient-centered counseling are paramount when selecting these intensive combination regimens in clinical practice. A summary of efficacy and safety information for each trial is available in **Table 2**.

**Table 2:** Summary of demographic characteristics, efficacy, and safety profile of clinical trials evaluating combination therapy for advanced *EGFR*-mutated NSCLC.

Trial	FLAURA	MARIPOSA	FLAURA 2
Tested treatment	Osimertinib monotherapy	Amivantamab + Lazertinib	Osimertinib + Chemotherapy
Number (n) of individuals	279	429	279
Median age (range) - years	64 (28-85)	64 (25-88)	61 (26-83)
Male (%)	36%	36%	38%
Adenocarcinoma (%)	99%	97%	99%
EGFR Exon 19 deletion (%)	63%	60%	61%
EGFR L858R (%)	35%	40%	38%
CNS metastasis (%)	19%	41%	42%
Median PFS	18.9 months	23.7 months	25.5 months
Median OS	38.6 months	3-year OS: 60% vs. 51% (HR 0.75; p=0.005)	47.5 months
Grade 3> adverse event rate	31%	80%	64%
Adverse events reported in over 50% of patients	Acne/Rash (58%), diarrhea (58%)	Paronychia (68%), IRRs (63%), acne/rash (62%)	–
Adverse events reported in over 30% of patients	Dry skin (36%) and paronychia (35%)	Hypoalbuminemia (48%), alanine aminotransferase (ALT) elevation (36%), peripheral edema (36%)	Anemia (46%), diarrhea (43%), nausea (43%)

## 5 Prophylaxis, preventive measures, and monitoring

First-generation EGFR tyrosine kinase inhibitors (EGFR-TKIs), including gefitinib and erlotinib, are associated predominantly with mild-to-moderate cutaneous and gastrointestinal toxicities. These adverse events commonly necessitate supportive and prophylactic measures, including emollients, sunscreen, and oral doxycycline or minocycline to prevent and mitigate acneiform rash, as well as loperamide for diarrhea management (23).

Third-generation EGFR-TKIs, such as osimertinib and lazertinib, do not require standardized prophylactic medications before treatment initiation. Instead, current clinical practice emphasizes risk-based assessment and close monitoring. The most frequently employed preventive interventions target dermatologic toxicity, including the routine use of emollients and sunscreen and, if necessary, early initiation of topical corticosteroids or antibiotics at a low threshold. Dietary counseling is recommended, and loperamide can be prescribed when clinically indicated (24). There is a recommendation of baseline and periodic electrocardiograms (EKGs) for patients with risk factors for QT interval prolongation or concomitant use of QT-prolonging medications (25).

In the phase III MARIPOSA trial, several toxicities were observed more frequently with amivantamab plus lazertinib than with osimertinib, reflecting combined inhibition of the EGFR and MET pathways and necessitating more extensive prophylactic and monitoring strategies. In the clinical trial protocol, prophylactic anticoagulation was mandatory during the first 4 months of treatment to mitigate the high rate of venous thromboembolic events (VTEs), which reached 37% (17). In real-world clinical practice and according to current regulatory drug labels, routine universal anticoagulation is not mandatory; instead, clinicians are advised to perform a careful, individualized risk-benefit assessment to guide the implementation of VTE prophylaxis, particularly during the initial months of highest risk. Oral dexamethasone administered 2 days prior to the first dose is recommended to mitigate infusion-related reactions (IRRs), and the initial infusions should be closely monitored. Additional dermatologic prophylaxis, including sunscreen use, oral doxycycline or minocycline, topical clindamycin applied to the scalp, chlorhexidine applied to the hands and feet, and ceramide-based, non-comedogenic moisturizers, is recommended to reduce the incidence and severity of EGFR-related skin toxicities (15).

Some commonly reported adverse effects of amivantamab plus lazertinib do not require continuous prophylactic measures but should be closely monitored for quick detection and management, including peripheral neuropathy, peripheral edema, hypoalbuminemia, ocular toxicities (e.g., dry eye, keratitis, blurred vision), pyogenic granuloma–like lesions (often periungual and associated with paronychia), and anogenital ulcers (17, 19, 26). Peripheral neuropathy management relies on early detection through routine neurologic assessment, and supportive measures (e.g., duloxetine or gabapentin for symptomatic relief) may be used when clinically indicated. Peripheral edema management is supportive and includes limb elevation, compression, lymphatic drainage massage, and, when appropriate, restriction of sodium intake. Hypoalbuminemia should be monitored with periodic laboratory assessment, and management consists of observation and correction of contributing factors, such as a low-protein diet, rather than prophylaxis (26). Ocular toxicities require baseline and routine ophthalmologic assessments to enable early referral to ophthalmology for persistent symptoms (15, 26). Pyogenic granuloma–like lesions require dermatologic management with topical corticosteroids, antiseptic care, and, if necessary, chemical cauterization, cryotherapy, or surgical treatment (26, 27). Although less common (generally <5%), anogenital ulcers are a distinctive EGFR-related mucocutaneous toxicity. Management includes local wound care, topical corticosteroids, and temporary treatment interruption for grade ≥2 lesions. Proactive monitoring, early supportive care, and adherence to dose-modification algorithms constitute the core management strategy for these toxicities (15, 26).

Chemotherapeutic regimens used in the FLAURA 2 trial include pemetrexed in combination with either cisplatin or carboplatin. Pemetrexed is associated with hematologic, gastrointestinal, renal, and cutaneous toxicities and carries a low to moderate emetogenic risk. Cisplatin is highly emetogenic, nephrotoxic, and associated with significant electrolyte wasting. Preventive strategies require optimized antiemetic prophylaxis, in addition to aggressive pre- and post-infusion intravenous hydration. Carboplatin is primarily associated with myelosuppression - most notably thrombocytopenia - and has moderate emetogenic potential. Preventive measures include antiemetic prophylaxis and routine monitoring of the complete blood count throughout treatment (28).

Although the phase III PALOMA-3 study was conducted in a refractory/post-progression setting (patients whose disease progressed after osimertinib and platinum-based chemotherapy), its findings regarding the subcutaneous administration of amivantamab are highly relevant to the clinical feasibility of first-line combination therapies (24). In PALOMA-3, subcutaneous amivantamab in combination with lazertinib demonstrated pharmacokinetic non-inferiority to the intravenous formulation, with comparable efficacy, significantly improved convenience (administration time of less than 5 minutes vs. several hours), and a dramatic reduction in infusion-related reactions (IRRs) (24). Consequently, the subcutaneous formulation is increasingly being integrated into first-line clinical protocols to improve safety and patient care.

## 6 Multidisciplinary approach to severe or complex toxicities

The clinical management of *EGFR*-mutated NSCLC patients involves a coordinated, risk-adapted approach. In routine clinical practice, the oncology care team directly manages the majority of mild-to-moderate (Grade 1–2) treatment-related toxicities through standard supportive care protocols, patient education, and established dose-modification guidelines, without requiring immediate specialist referral. However, a structured multidisciplinary approach becomes essential for the management of severe (Grade ≥ 3) or highly complex adverse events, which is critical to optimize patient safety, support treatment adherence, and maintain quality of life (29).

Dermatology consultation plays a crucial role in severe, refractory, or atypical EGFR-TKI and amivantamab-related cutaneous toxicities, such as severe paronychia (Grade ≥ 3), persistent acneiform dermatitis requiring systemic retinoids, or suspected secondary skin infections, rather than routine mild dry skin or rash (15, 26, 27). Similarly, nephrology involvement is particularly important with cisplatin and pemetrexed therapy. While routine nephrology consultation is not standard practice for patients receiving platinum-based regimens, it is highly recommended for those with pre-existing renal impairment, severe chronic kidney disease, or those experiencing acute, severe therapy-induced renal toxicity (30, 31).

Cardiology collaboration is highly relevant for patients with pre-existing cardiovascular risk factors who develop Grade 3–4 QT interval prolongation or clinical signs of cardiomyopathy during third-generation EGFR-TKI therapy (12, 25). Ophthalmologic referral remains standard for persistent ocular symptoms, dry eye, or suspected keratitis (15). Finally, neurology consultation is indicated for the assessment of clinically significant peripheral neuropathy observed with cisplatin or carboplatin regimens (30). By focusing multidisciplinary collaboration on severe or complex toxicities, clinical workflows are optimized, ensuring that specialized medical resources are deployed where they provide the greatest clinical benefit.

## 7 Shared decision-making: the trade-off between effectiveness and adverse events/side effects

In clinical trials, PFS, defined as the time from randomization to tumor progression or death, has become a commonly used primary outcome for drug regulatory decisions and clinical guideline recommendations by the FDA (32, 33) and the European Medicines Agency (EMA) (34). However, given the high burden of NSCLC and the fact that almost 80% of patients are diagnosed at stage III or IV, when the tumor has already metastasized and surgical resection is not possible, treatment choice takes into consideration several other characteristics beyond PFS, such as modes of administration, time dedicated to treatment, and the need for prophylactic and other concurrent treatments that may affect quality of life (35).

Although the clinical progression of cancer is distressing, in the palliative treatment setting, most patients do not prefer treatments that worsen clinical symptoms, prolong time in the health care system, or otherwise diminish quality of life, even with substantial gains in PFS (36). In a discrete choice experiment (DCE) in which lung cancer patients were asked about what drove their therapy choices, a therapy’s ability to reduce tumor-related symptoms (e.g., pain, coughing, and shortness of breath) was the most influential factor in treatment decision-making (37).

A discrete-choice simulation study involving patients with incurable solid tumors undergoing systemic therapy evaluated patient willingness to accept additional treatment-related toxicities and clinical visits for marginal survival gains (39). The study revealed that 51% of participants would not accept any additional toxicity for any magnitude of progression-free or overall survival gain, prioritizing immediate quality of life and emotional well-being (39). Although specific first-line discrete choice experiments comparing oral targeted monotherapy directly against intensive intravenous combinations (e.g., amivantamab plus lazertinib or chemotherapy combinations) are still limited, these general preference data are highly informative. They provide a clear behavioral rationale for why many patients and clinicians, in clinical practice, prefer first-line osimertinib monotherapy - an oral option with a highly manageable toxicity profile and minimal hospital-bound monitoring burden - over highly intensive combination regimens, unless high-risk clinical factors (e.g., extensive baseline CNS or liver metastases) warrant chemotherapy intensification or dual blockade.

A recent cross-sectional online survey found that patients, care partners, and physicians rate OS as the most important treatment attribute. Quality of life, ranked second by patients and care partners, was ranked fourth by clinicians (38).

Studies like these highlight the importance of having the full spectrum of information for the decision-making process, including the risk for side effects and adverse events, prophylactic treatments, other parallel treatments, and the required physicians of multiple specialties. Additionally, patient characteristics, such as advanced age (39), site of metastasis (40, 41), and functional capacity (42) (usually classified by the Eastern Cooperative Oncology Group [ECOG] Performance Status scale), vary and are highly influential in shaping each individual’s priorities.

Notably, rather than being competing or mutually exclusive considerations, PFS and quality of life represent complementary endpoints in the management of advanced *EGFR*-mutated NSCLC. Prolonging PFS through effective first-line therapy may delay disease-related symptomatic progression, and thereby help preserve functional status and maintain or improve patient-reported quality of life (12, 17, 20). Therefore, survival outcomes and quality of life should both be integrated into patient-centered shared decision-making conversations, balancing the magnitude of clinical benefit against the required monitoring burden and toxicity profiles of each therapeutic regimen (35–37).

## 8 Discussion

The therapeutic landscape for *EGFR*-mutated NSCLC has evolved rapidly over the past decade, shifting from sequential single-agent tyrosine kinase inhibition toward increasingly intensive combination strategies (43). This narrative review synthesizes current evidence on first-line treatments for advanced *EGFR*-mutated NSCLC, with particular emphasis on efficacy–toxicity trade-offs, real-world feasibility, and patient-centered considerations.

Osimertinib monotherapy is most commonly used as a first-line treatment because the FLAURA trial demonstrated superior PFS and improved central nervous system (CNS) control compared with first-generation EGFR-TKIs, along with a more favorable safety profile (6, 12). The durability of benefit, manageable adverse events, oral administration, and limited need for prophylactic interventions make osimertinib particularly attractive in real-world settings, where access to multidisciplinary supportive care may be heterogeneous.

More recently, combination strategies have challenged osimertinib monotherapy as the default first-line option. The MARIPOSA trial demonstrated clinically meaningful benefits in PFS and OS with amivantamab plus lazertinib compared with osimertinib, supporting the biological rationale for dual EGFR and MET inhibition as a viable first-line treatment for the most common *EGFR* mutation. However, this benefit was accompanied by increased rates of grade ≥3 adverse events, infusion-related reactions, venous thromboembolism, and treatment discontinuation (17, 27). These findings highlight that the efficacy gains of combination therapy are inseparable from increased toxicity, treatment burden, and healthcare system utilization (44).

Collectively, the data reviewed illustrate that while newer regimens consistently extend PFS and OS, they do so at the cost of substantially increased treatment complexity and toxicity, reinforcing the need for individualized, shared decision-making. It is critical to clarify that this increase in treatment-related adverse events is not driven by newer TKIs as monotherapy, which actually demonstrate a more favorable safety profile than older-generation agents, but rather by the transition to intensive first-line combination regimens (such as amivantamab plus lazertinib or osimertinib plus platinum-pemetrexed chemotherapy).

Across all regimens reviewed, a critical question is whether incremental gains in PFS and OS justify the added toxicity, impact on quality of life, and increased cost of treatment. From a regional perspective, Brazil and South America face additional challenges, including variable access to molecular testing, delayed diagnosis, disparities between public and private healthcare systems, and limited availability of newer agents. While international clinical trials define efficacy under controlled conditions, translating this into routine practice requires contextual adaptation, as specific biomarkers associated with improved benefit have yet to be identified (45).

The Sociedade Brasileira de Oncologia Clínica (SBOC) is the principal professional society representing medical oncologists in Brazil. It plays a central role in issuing evidence-based consensus statements adapted to the Brazilian regulatory and healthcare context. For advanced NSCLC harboring sensitizing *EGFR* mutations (exon 19 deletion or L858R), SBOC-aligned recommendations prioritize comprehensive molecular testing and endorse targeted therapy as the first-line treatment. Within the Brazilian clinical context and according to the SBOC consensus (7), osimertinib monotherapy is recognized as the preferred first-line standard of care. This preference is primarily driven by its oral feasibility, highly favorable safety profile, and broader (although limited) reimbursement access compared to newer combinations. While international guidelines, such as the NCCN (15), endorse osimertinib monotherapy, osimertinib plus chemotherapy, and amivantamab plus lazertinib as equivalent Category 1 first-line options, the intensive combination regimens face substantial financial, logistical, and administrative barriers to clinical implementation in Brazil. These barriers are particularly pronounced within the public healthcare system (SUS), where the lack of CONITEC incorporation restricts first-line treatment to older-generation agents, whereas the private sector benefits from mandatory ANS coverage for these newly approved regimens.

However, access to newer therapies, such as amivantamab and lazertinib, remains limited, being implemented only in the private sector, creating significant disparities in care. Additionally, the availability of comprehensive molecular testing varies substantially across regions, with reference centers concentrated in major urban areas, which can lead to diagnostic delays that may compromise the optimal timing of targeted therapy initiation. The infrastructure for managing complex toxicities, particularly the multidisciplinary support required for combination regimens, also exhibits heterogeneity, with more robust support in specialized centers. These factors reinforce the need for strategies adapted to the local context, prioritizing not only clinical efficacy but also implementation feasibility and sustainability within the Brazilian healthcare system.

Furthermore, understanding the mechanisms of acquired resistance to first-line third-generation EGFR-TKIs is crucial for guiding subsequent therapeutic sequencing. Acquired resistance generally occurs through on-target or off-target genomic alterations. On-target mechanisms include the loss of the T790M mutation (if present) or the development of secondary *EGFR* mutations, most notably the C797S mutation in exon 20, which hinders osimertinib binding. Off-target resistance mechanisms bypass EGFR inhibition through the activation of alternative bypass signaling pathways, predominantly *MET* gene amplification (occurring in approximately 15% of cases), *HER2* amplification, or *BRAF* mutations. Additionally, histologic transformation to small-cell lung cancer (SCLC) or other histologies represents a well-documented resistance mechanism occurring in 3% to 15% of patients. Identifying these specific resistance pathways via tissue re-biopsy at the time of disease progression is increasingly vital to guide subsequent personalized therapeutic choices, such as dual EGFR/MET inhibition or chemotherapy-based regimens (46–48).

In conclusion, the central challenge is no longer the lack of effective treatments but rather determining how to deploy them. Future research should prioritize not only survival endpoints, but also patient-reported outcomes, real-world effectiveness, and health system sustainability. Ultimately, optimal care for *EGFR*-mutated NSCLC will depend on aligning biological rationale, clinical evidence, patient values, and regional realities into truly personalized treatment strategies (**Figure 3**).

**Figure 3:**
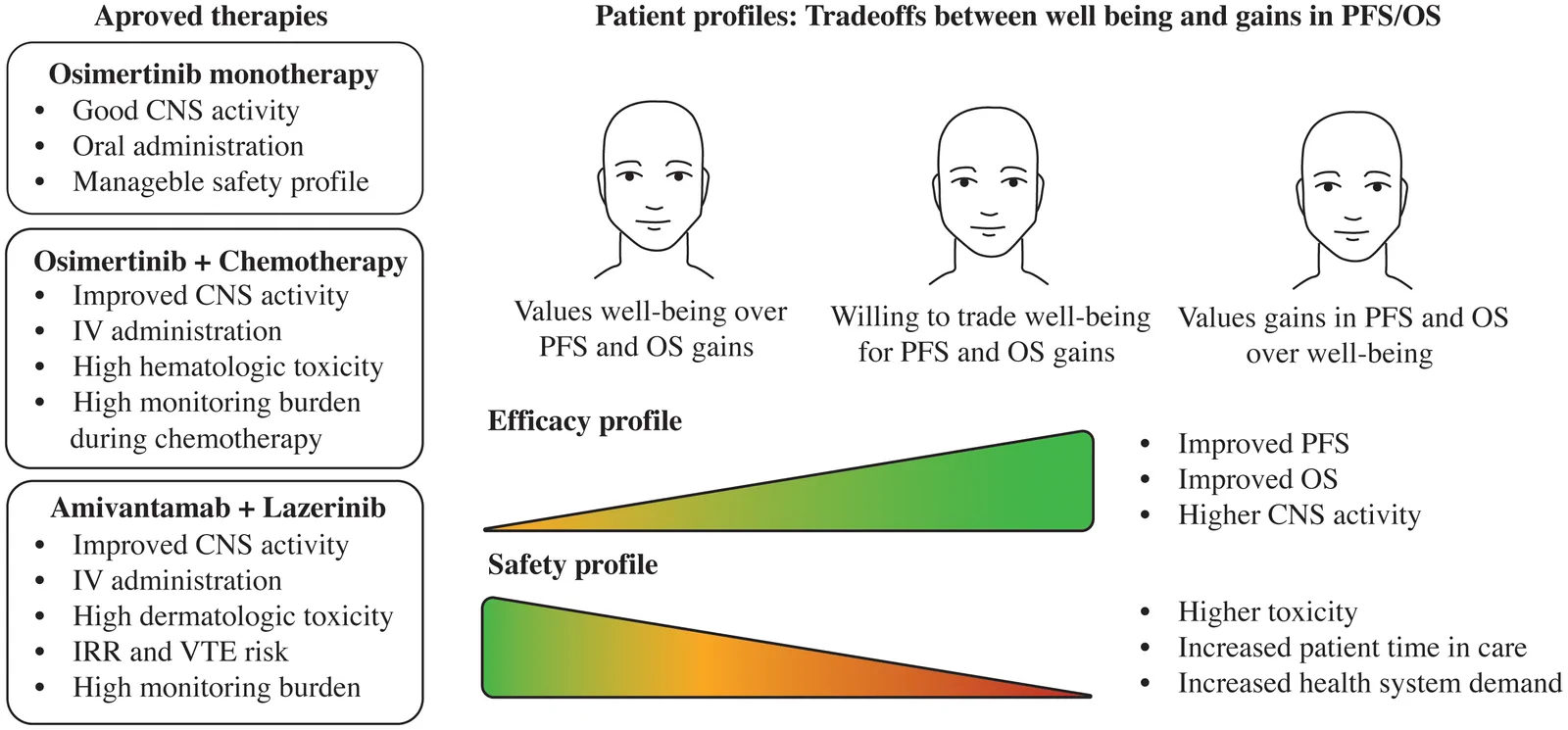
Schematics summarizing the parameters taken into consideration when choosing a personalized treatment strategy for advanced *EGFR*-mutated NSCLC.
